# 8-Hy­droxy-2-methyl­quinolinium dibromido(2-methyl­quinolin-8-olato-κ^2^ *N*,*O*)zincate acetonitrile mono­solvate

**DOI:** 10.1107/S160053681103234X

**Published:** 2011-08-27

**Authors:** Ezzatollah Najafi, Mostafa M. Amini, Seik Weng Ng

**Affiliations:** a Department of Chemistry, General Campus, Shahid Beheshti University, Tehran 1983963113, Iran; b Department of Chemistry, University of Malaya, 50603 Kuala Lumpur, Malaysia; c Chemistry Department, Faculty of Science, King Abdulaziz University, PO Box 80203 Jeddah, Saudi Arabia

## Abstract

The reaction of 2-methyl-8-hy­droxy­quinoline and zinc bromide in acetonitrile affords the title solvated salt, (C_10_H_10_NO)[ZnBr_2_(C_10_H_8_NO)]·CH_3_CN, in which the Zn^II^ ion is coordinated by a *N*,*O*-chelating 2-methyl­quinolin-8-olate ligand and two bromide ligands in a distorted tetra­hedral geometry. The cation is linked to the anion by an O—H⋯O hydrogen bond and the quinolinium H atom forms an inter­molecular N—H⋯N hydrogen bond with the acetonitrile solvent mol­ecule.

## Related literature

For the crystal structure of 8-hy­droxy-2-methyl­quinolinium dichlorido(2-methyl­quinolin-8-olato)zincate acetonitrile disolvate, see: Najafi *et al.* (2011 ▶). For the crystal structures of related methanol solvates, see: Najafi *et al.* (2010*a* ▶, 2010*b* ▶); Sattarzadeh *et al.* (2009 ▶).
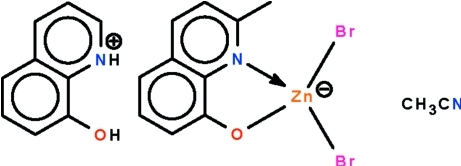


## Experimental

### 

#### Crystal data

(C_10_H_10_NO)[ZnBr_2_(C_10_H_8_NO)]·C_2_H_3_N
*M* *_r_* = 584.61
Triclinic, 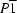

*a* = 7.1870 (3) Å
*b* = 9.7795 (5) Å
*c* = 16.2520 (7) Å
α = 86.159 (4)°
β = 80.775 (4)°
γ = 85.098 (4)°
*V* = 1121.73 (9) Å^3^
*Z* = 2
Mo *K*α radiation
μ = 4.68 mm^−1^
*T* = 100 K
0.25 × 0.20 × 0.15 mm

#### Data collection

Agilent SuperNova Dual diffractometer with an Atlas detector
Absorption correction: multi-scan (*CrysAlis PRO*; Agilent, 2010 ▶) *T*_min_ = 0.387, *T*_max_ = 0.540
8710 measured reflections
4970 independent reflections
4197 reflections with *I* > 2σ(*I*)
*R*_int_ = 0.033

#### Refinement

*R*[*F* ^2^ > 2σ(*F* ^2^)] = 0.031
*wR*(*F* ^2^) = 0.065
*S* = 1.01
4970 reflections
282 parameters
2 restraints
H atoms treated by a mixture of independent and constrained refinement
Δρ_max_ = 0.54 e Å^−3^
Δρ_min_ = −0.86 e Å^−3^

### 

Data collection: *CrysAlis PRO* (Agilent, 2010 ▶); cell refinement: *CrysAlis PRO*; data reduction: *CrysAlis PRO*; program(s) used to solve structure: *SHELXS97* (Sheldrick, 2008 ▶); program(s) used to refine structure: *SHELXL97* (Sheldrick, 2008 ▶); molecular graphics: *X-SEED* (Barbour, 2001 ▶); software used to prepare material for publication: *publCIF* (Westrip, 2010 ▶).

## Supplementary Material

Crystal structure: contains datablock(s) global, I. DOI: 10.1107/S160053681103234X/lh5306sup1.cif

Structure factors: contains datablock(s) I. DOI: 10.1107/S160053681103234X/lh5306Isup2.hkl

Additional supplementary materials:  crystallographic information; 3D view; checkCIF report

## Figures and Tables

**Table 1:** Hydrogen-bond geometry (Å, °)

*D*—H⋯*A*	*D*—H	H⋯*A*	*D*⋯*A*	*D*—H⋯*A*
O2—H2⋯O1	0.84 (1)	1.73 (1)	2.561 (2)	173 (4)
N2—H1⋯N3	0.88 (1)	2.08 (1)	2.943 (3)	171 (3)

**Fig. 1.**
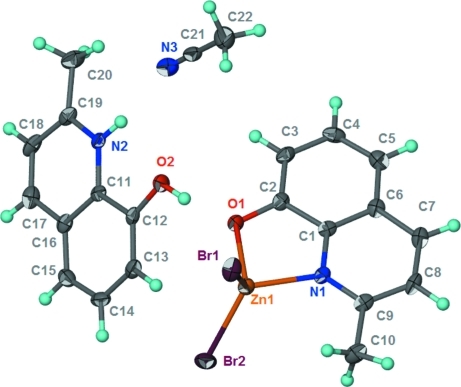
Displacement ellipsoid plot (Barbour, 2001) of (C10H10NO)[ZnBr2(C10H8NO)].CH3CN at the 70% probability level; hydrogen atoms are drawn as spheres of arbitrary radius.

## supplementary crystallographic information

### Comment

We have synthesized methanol-solvated 8-hydroxy-2-methylquinolinium dihalo(2-methylquinolin-8-olato)zincates(II) by the direct reaction of the zinc halide and 8-hydroxy-2-methylquinolin in methanol. The salts have the Zn^II^ atom in a tetrahedral geometry, and the ion-pairs are linked to the solvent molecules by hydrogen bonds (Najafi *et al.*, 2010*a*; Najafi *et al.*, 2010*b*; Sattarzadeh *et al.*, 2009). In this study we used acetonitrile as a solvent. In a previous study, the reaction of zinc chloride and the quinoline in acetonitrile yielded the disolvated salt (Najafi *et al.*, 2011). In the present study, zinc dibromide gave a mono-solvated salt (Fig. 1). In (C_10_H_10_NO)[ZnBr_2_(C_10_H_8_NO)]^.^CH_3_CN, the metal in the anion is *N*,*O*-chelated by the deprotonated ligand and it exists in a distorted tetrahedral geometry. The cation is linked to the anion by an O–H···O hydrogen bond and the quinolinium H atom forms a hydrogen bond with the solvent molecule (Table 1).

### Experimental

Zinc chloride (0.23 g, 0.75 mmol) and 2-methyl-8-hydroxyquinoline (0.24 g, 1.5 mmol) were loaded into a convection tube and the tube was filled with acetonitrile and kept at 333 K. Yellow crystals were collected from the side arm after several days.

### Refinement

Carbon-bound H-atoms were placed in calculated positions [C—H 0.95 to 0.98 Å, *U*_iso_(H) 1.2 to 1.5*U*_eq_(C)] and were included in the refinement in the riding model approximation. The N and O bound H atoms were located in a difference Fourier map, and were refined with distance restraints of N–H 0.88±0.01, O–H 0.84±0.01 Å; their temperature factors were refined. The (2 - 2 7) and (0 1 1) were removed.

### Crystal data

**Table:**

(C_10_H_10_NO)[ZnBr_2_(C_10_H_8_NO)]·C_2_H_3_N	*Z* = 2
*M**_r_* = 584.61	*F*(000) = 580
Triclinic, *P*1	*D*_x_ = 1.731 Mg m^−^^3^
Hall symbol: -P 1	Mo *K*α radiation, λ = 0.71073 Å
*a* = 7.1870 (3) Å	Cell parameters from 4618 reflections
*b* = 9.7795 (5) Å	θ = 2.4–29.1°
*c* = 16.2520 (7) Å	µ = 4.68 mm^−^^1^
α = 86.159 (4)°	*T* = 100 K
β = 80.775 (4)°	Block, yellow
γ = 85.098 (4)°	0.25 × 0.20 × 0.15 mm
*V* = 1121.73 (9) Å^3^	

### Data collection

**Table:**

Agilent SuperNova Dual diffractometer with an Atlas detector	4970 independent reflections
Radiation source: SuperNova (Mo) X-ray Source	4197 reflections with *I* > 2σ(*I*)
Mirror	*R*_int_ = 0.033
Detector resolution: 10.4041 pixels mm^-1^	θ_max_ = 27.5°, θ_min_ = 2.5°
ω scans	*h* = −9→9
Absorption correction: multi-scan (*CrysAlis PRO*; Agilent, 2010)	*k* = −9→12
*T*_min_ = 0.387, *T*_max_ = 0.540	*l* = −20→21
8710 measured reflections	

### Refinement

**Table:**

Refinement on *F*^2^	Primary atom site location: structure-invariant direct methods
Least-squares matrix: full	Secondary atom site location: difference Fourier map
*R*[*F*^2^ > 2σ(*F*^2^)] = 0.031	Hydrogen site location: inferred from neighbouring sites
*wR*(*F*^2^) = 0.065	H atoms treated by a mixture of independent and constrained refinement
*S* = 1.01	*w* = 1/[σ^2^(*F*_o_^2^) + (0.0183*P*)^2^] where *P* = (*F*_o_^2^ + 2*F*_c_^2^)/3
4970 reflections	(Δ/σ)_max_ = 0.001
282 parameters	Δρ_max_ = 0.54 e Å^−^^3^
2 restraints	Δρ_min_ = −0.86 e Å^−^^3^

### Fractional atomic coordinates and isotropic or equivalent isotropic displacement parameters (Å^2^)

**Table:**

	*x*	*y*	*z*	*U*_iso_*/*U*_eq_	
Br1	0.88168 (4)	0.83050 (3)	0.860009 (16)	0.02244 (8)	
Br2	0.50073 (4)	0.95447 (3)	0.711001 (16)	0.02404 (8)	
Zn1	0.61597 (4)	0.77959 (3)	0.800839 (17)	0.01579 (8)	
O1	0.6552 (2)	0.59396 (18)	0.75476 (10)	0.0164 (4)	
O2	0.7760 (2)	0.49663 (19)	0.61189 (10)	0.0180 (4)	
H2	0.735 (4)	0.535 (3)	0.6565 (12)	0.051 (11)*	
N1	0.4256 (3)	0.6890 (2)	0.88999 (12)	0.0138 (5)	
N2	0.8330 (3)	0.3677 (2)	0.46831 (12)	0.0137 (5)	
H1	0.853 (4)	0.323 (3)	0.5148 (10)	0.026 (8)*	
N3	0.9248 (3)	0.1939 (2)	0.61350 (15)	0.0268 (6)	
C1	0.4381 (3)	0.5504 (3)	0.87876 (14)	0.0135 (5)	
C2	0.5632 (3)	0.5020 (3)	0.80728 (14)	0.0149 (5)	
C3	0.5768 (4)	0.3633 (3)	0.79535 (15)	0.0173 (6)	
H3	0.6582	0.3288	0.7481	0.021*	
C4	0.4733 (4)	0.2716 (3)	0.85143 (15)	0.0182 (6)	
H4	0.4860	0.1765	0.8411	0.022*	
C5	0.3544 (3)	0.3161 (3)	0.92073 (16)	0.0187 (6)	
H5	0.2857	0.2527	0.9581	0.022*	
C6	0.3357 (3)	0.4576 (3)	0.93569 (15)	0.0156 (5)	
C7	0.2193 (3)	0.5165 (3)	1.00595 (15)	0.0179 (6)	
H7	0.1498	0.4591	1.0471	0.022*	
C8	0.2076 (3)	0.6539 (3)	1.01421 (15)	0.0181 (6)	
H8	0.1275	0.6922	1.0606	0.022*	
C9	0.3124 (3)	0.7408 (3)	0.95511 (15)	0.0156 (6)	
C10	0.3020 (4)	0.8932 (3)	0.96312 (16)	0.0222 (6)	
H10A	0.4281	0.9263	0.9462	0.033*	
H10B	0.2555	0.9144	1.0213	0.033*	
H10C	0.2155	0.9387	0.9271	0.033*	
C11	0.7546 (3)	0.5007 (3)	0.46969 (15)	0.0134 (5)	
C12	0.7190 (3)	0.5680 (3)	0.54621 (15)	0.0143 (5)	
C13	0.6326 (3)	0.6992 (3)	0.54599 (16)	0.0173 (6)	
H13	0.6071	0.7463	0.5963	0.021*	
C14	0.5817 (3)	0.7643 (3)	0.47216 (16)	0.0190 (6)	
H14	0.5201	0.8542	0.4740	0.023*	
C15	0.6185 (3)	0.7019 (3)	0.39779 (16)	0.0182 (6)	
H15	0.5836	0.7480	0.3486	0.022*	
C16	0.7093 (3)	0.5677 (3)	0.39514 (15)	0.0159 (6)	
C17	0.7563 (3)	0.4941 (3)	0.32169 (16)	0.0202 (6)	
H17	0.7326	0.5375	0.2699	0.024*	
C18	0.8347 (3)	0.3626 (3)	0.32364 (16)	0.0200 (6)	
H18	0.8650	0.3152	0.2735	0.024*	
C19	0.8711 (3)	0.2964 (3)	0.39969 (16)	0.0173 (6)	
C20	0.9466 (4)	0.1505 (3)	0.40586 (17)	0.0248 (6)	
H20A	0.9691	0.1260	0.4631	0.037*	
H20B	1.0657	0.1380	0.3673	0.037*	
H20C	0.8548	0.0913	0.3913	0.037*	
C21	0.9679 (4)	0.1787 (3)	0.67766 (18)	0.0220 (6)	
C22	1.0230 (4)	0.1609 (3)	0.76012 (17)	0.0322 (7)	
H22A	1.1477	0.1105	0.7564	0.048*	
H22B	0.9301	0.1090	0.7976	0.048*	
H22C	1.0285	0.2512	0.7820	0.048*	

### Atomic displacement parameters (Å^2^)

**Table:**

	*U*^11^	*U*^22^	*U*^33^	*U*^12^	*U*^13^	*U*^23^
Br1	0.02081 (15)	0.02701 (17)	0.02033 (14)	−0.00692 (12)	−0.00113 (11)	−0.00586 (12)
Br2	0.03621 (17)	0.01423 (15)	0.02002 (14)	0.00516 (12)	−0.00265 (12)	−0.00189 (11)
Zn1	0.01904 (16)	0.01350 (17)	0.01443 (15)	−0.00166 (13)	−0.00014 (12)	−0.00329 (12)
O1	0.0210 (9)	0.0138 (10)	0.0130 (8)	0.0004 (8)	0.0010 (7)	−0.0027 (7)
O2	0.0249 (10)	0.0167 (10)	0.0112 (9)	0.0011 (8)	−0.0001 (8)	−0.0020 (8)
N1	0.0138 (10)	0.0161 (12)	0.0120 (10)	−0.0006 (9)	−0.0024 (8)	−0.0043 (9)
N2	0.0137 (11)	0.0139 (12)	0.0126 (11)	−0.0010 (9)	0.0015 (9)	−0.0028 (9)
N3	0.0290 (14)	0.0192 (14)	0.0296 (14)	0.0031 (11)	0.0006 (11)	−0.0007 (11)
C1	0.0136 (12)	0.0148 (14)	0.0136 (12)	−0.0001 (10)	−0.0059 (10)	−0.0046 (10)
C2	0.0157 (13)	0.0187 (15)	0.0109 (12)	−0.0003 (11)	−0.0038 (10)	−0.0030 (11)
C3	0.0197 (13)	0.0191 (15)	0.0132 (12)	0.0042 (11)	−0.0033 (11)	−0.0065 (11)
C4	0.0245 (14)	0.0115 (14)	0.0210 (13)	−0.0003 (11)	−0.0106 (11)	−0.0032 (11)
C5	0.0190 (13)	0.0187 (15)	0.0202 (13)	−0.0052 (12)	−0.0068 (11)	0.0006 (11)
C6	0.0132 (12)	0.0201 (15)	0.0147 (12)	−0.0020 (11)	−0.0051 (10)	−0.0016 (11)
C7	0.0144 (13)	0.0252 (16)	0.0146 (12)	−0.0035 (12)	−0.0025 (10)	−0.0016 (11)
C8	0.0125 (12)	0.0279 (16)	0.0139 (12)	−0.0004 (12)	0.0000 (10)	−0.0067 (12)
C9	0.0143 (13)	0.0181 (15)	0.0160 (13)	−0.0004 (11)	−0.0059 (10)	−0.0061 (11)
C10	0.0224 (14)	0.0221 (16)	0.0225 (14)	0.0008 (12)	−0.0021 (12)	−0.0095 (12)
C11	0.0081 (12)	0.0136 (13)	0.0183 (13)	−0.0027 (10)	0.0001 (10)	−0.0015 (11)
C12	0.0125 (12)	0.0157 (14)	0.0139 (12)	−0.0042 (11)	0.0025 (10)	−0.0033 (11)
C13	0.0168 (13)	0.0160 (14)	0.0185 (13)	−0.0049 (11)	0.0028 (11)	−0.0051 (11)
C14	0.0153 (13)	0.0125 (14)	0.0279 (15)	0.0013 (11)	−0.0006 (11)	−0.0009 (11)
C15	0.0175 (13)	0.0172 (15)	0.0194 (13)	−0.0026 (11)	−0.0026 (11)	0.0034 (11)
C16	0.0113 (12)	0.0204 (15)	0.0162 (13)	−0.0028 (11)	−0.0007 (10)	−0.0030 (11)
C17	0.0175 (14)	0.0276 (17)	0.0162 (13)	−0.0050 (12)	−0.0024 (11)	−0.0033 (12)
C18	0.0181 (14)	0.0263 (16)	0.0161 (13)	−0.0008 (12)	0.0003 (11)	−0.0133 (12)
C19	0.0132 (13)	0.0177 (15)	0.0211 (14)	−0.0024 (11)	0.0005 (11)	−0.0066 (11)
C20	0.0282 (15)	0.0195 (16)	0.0263 (15)	0.0037 (13)	−0.0029 (12)	−0.0086 (12)
C21	0.0205 (14)	0.0115 (14)	0.0302 (16)	0.0020 (11)	0.0054 (13)	0.0000 (12)
C22	0.0312 (17)	0.036 (2)	0.0251 (15)	0.0058 (15)	0.0005 (13)	0.0072 (14)

### Geometric parameters (Å, °)

**Table:**

Br1—Zn1	2.3738 (4)	C9—C10	1.500 (4)
Br2—Zn1	2.3566 (4)	C10—H10A	0.9800
Zn1—O1	1.9907 (18)	C10—H10B	0.9800
Zn1—N1	2.0386 (19)	C10—H10C	0.9800
O1—C2	1.339 (3)	C11—C16	1.409 (3)
O2—C12	1.336 (3)	C11—C12	1.423 (3)
O2—H2	0.837 (10)	C12—C13	1.377 (4)
N1—C9	1.328 (3)	C13—C14	1.406 (4)
N1—C1	1.373 (3)	C13—H13	0.9500
N2—C19	1.334 (3)	C14—C15	1.369 (4)
N2—C11	1.373 (3)	C14—H14	0.9500
N2—H1	0.875 (10)	C15—C16	1.415 (4)
N3—C21	1.131 (3)	C15—H15	0.9500
C1—C6	1.416 (3)	C16—C17	1.415 (4)
C1—C2	1.431 (3)	C17—C18	1.360 (4)
C2—C3	1.375 (4)	C17—H17	0.9500
C3—C4	1.406 (4)	C18—C19	1.408 (4)
C3—H3	0.9500	C18—H18	0.9500
C4—C5	1.372 (3)	C19—C20	1.485 (4)
C4—H4	0.9500	C20—H20A	0.9800
C5—C6	1.412 (4)	C20—H20B	0.9800
C5—H5	0.9500	C20—H20C	0.9800
C6—C7	1.426 (3)	C21—C22	1.453 (4)
C7—C8	1.353 (4)	C22—H22A	0.9800
C7—H7	0.9500	C22—H22B	0.9800
C8—C9	1.407 (4)	C22—H22C	0.9800
C8—H8	0.9500		
			
O1—Zn1—N1	83.97 (8)	C9—C10—H10C	109.5
O1—Zn1—Br2	114.56 (5)	H10A—C10—H10C	109.5
N1—Zn1—Br2	117.98 (6)	H10B—C10—H10C	109.5
O1—Zn1—Br1	111.06 (5)	N2—C11—C16	119.2 (2)
N1—Zn1—Br1	109.80 (6)	N2—C11—C12	119.6 (2)
Br2—Zn1—Br1	115.446 (16)	C16—C11—C12	121.2 (2)
C2—O1—Zn1	110.82 (14)	O2—C12—C13	126.2 (2)
C12—O2—H2	112 (2)	O2—C12—C11	115.8 (2)
C9—N1—C1	120.2 (2)	C13—C12—C11	118.0 (2)
C9—N1—Zn1	130.70 (18)	C12—C13—C14	120.8 (2)
C1—N1—Zn1	108.86 (15)	C12—C13—H13	119.6
C19—N2—C11	123.8 (2)	C14—C13—H13	119.6
C19—N2—H1	116.0 (19)	C15—C14—C13	121.8 (3)
C11—N2—H1	120.1 (19)	C15—C14—H14	119.1
N1—C1—C6	122.2 (2)	C13—C14—H14	119.1
N1—C1—C2	117.1 (2)	C14—C15—C16	119.1 (2)
C6—C1—C2	120.7 (2)	C14—C15—H15	120.4
O1—C2—C3	123.9 (2)	C16—C15—H15	120.4
O1—C2—C1	118.5 (2)	C11—C16—C17	117.0 (2)
C3—C2—C1	117.6 (2)	C11—C16—C15	119.0 (2)
C2—C3—C4	121.5 (2)	C17—C16—C15	124.0 (2)
C2—C3—H3	119.2	C18—C17—C16	121.4 (3)
C4—C3—H3	119.2	C18—C17—H17	119.3
C5—C4—C3	121.6 (3)	C16—C17—H17	119.3
C5—C4—H4	119.2	C17—C18—C19	120.2 (2)
C3—C4—H4	119.2	C17—C18—H18	119.9
C4—C5—C6	119.0 (2)	C19—C18—H18	119.9
C4—C5—H5	120.5	N2—C19—C18	118.2 (2)
C6—C5—H5	120.5	N2—C19—C20	119.5 (2)
C1—C6—C5	119.5 (2)	C18—C19—C20	122.3 (2)
C1—C6—C7	116.0 (2)	C19—C20—H20A	109.5
C5—C6—C7	124.5 (2)	C19—C20—H20B	109.5
C8—C7—C6	120.2 (2)	H20A—C20—H20B	109.5
C8—C7—H7	119.9	C19—C20—H20C	109.5
C6—C7—H7	119.9	H20A—C20—H20C	109.5
C7—C8—C9	121.0 (2)	H20B—C20—H20C	109.5
C7—C8—H8	119.5	N3—C21—C22	179.3 (3)
C9—C8—H8	119.5	C21—C22—H22A	109.5
N1—C9—C8	120.4 (2)	C21—C22—H22B	109.5
N1—C9—C10	117.7 (2)	H22A—C22—H22B	109.5
C8—C9—C10	121.9 (2)	C21—C22—H22C	109.5
C9—C10—H10A	109.5	H22A—C22—H22C	109.5
C9—C10—H10B	109.5	H22B—C22—H22C	109.5
H10A—C10—H10B	109.5		
			
N1—Zn1—O1—C2	−7.93 (16)	C6—C7—C8—C9	−1.4 (4)
Br2—Zn1—O1—C2	−125.99 (14)	C1—N1—C9—C8	1.5 (3)
Br1—Zn1—O1—C2	101.00 (15)	Zn1—N1—C9—C8	−172.56 (17)
O1—Zn1—N1—C9	−178.6 (2)	C1—N1—C9—C10	−178.9 (2)
Br2—Zn1—N1—C9	−64.0 (2)	Zn1—N1—C9—C10	7.1 (3)
Br1—Zn1—N1—C9	71.1 (2)	C7—C8—C9—N1	−0.2 (4)
O1—Zn1—N1—C1	6.82 (15)	C7—C8—C9—C10	−179.8 (2)
Br2—Zn1—N1—C1	121.47 (14)	C19—N2—C11—C16	0.4 (4)
Br1—Zn1—N1—C1	−103.42 (15)	C19—N2—C11—C12	179.9 (2)
C9—N1—C1—C6	−1.1 (4)	N2—C11—C12—O2	3.6 (3)
Zn1—N1—C1—C6	174.10 (19)	C16—C11—C12—O2	−176.9 (2)
C9—N1—C1—C2	−179.9 (2)	N2—C11—C12—C13	−177.2 (2)
Zn1—N1—C1—C2	−4.7 (3)	C16—C11—C12—C13	2.2 (3)
Zn1—O1—C2—C3	−174.4 (2)	O2—C12—C13—C14	179.0 (2)
Zn1—O1—C2—C1	7.7 (3)	C11—C12—C13—C14	−0.1 (4)
N1—C1—C2—O1	−2.0 (3)	C12—C13—C14—C15	−1.2 (4)
C6—C1—C2—O1	179.2 (2)	C13—C14—C15—C16	0.4 (4)
N1—C1—C2—C3	−180.0 (2)	N2—C11—C16—C17	−2.8 (3)
C6—C1—C2—C3	1.2 (4)	C12—C11—C16—C17	177.8 (2)
O1—C2—C3—C4	−178.4 (2)	N2—C11—C16—C15	176.4 (2)
C1—C2—C3—C4	−0.5 (4)	C12—C11—C16—C15	−3.1 (3)
C2—C3—C4—C5	−0.2 (4)	C14—C15—C16—C11	1.7 (4)
C3—C4—C5—C6	0.2 (4)	C14—C15—C16—C17	−179.2 (2)
N1—C1—C6—C5	180.0 (2)	C11—C16—C17—C18	2.6 (4)
C2—C1—C6—C5	−1.3 (4)	C15—C16—C17—C18	−176.5 (2)
N1—C1—C6—C7	−0.5 (3)	C16—C17—C18—C19	0.0 (4)
C2—C1—C6—C7	178.3 (2)	C11—N2—C19—C18	2.2 (4)
C4—C5—C6—C1	0.6 (4)	C11—N2—C19—C20	−176.8 (2)
C4—C5—C6—C7	−179.0 (2)	C17—C18—C19—N2	−2.3 (4)
C1—C6—C7—C8	1.7 (4)	C17—C18—C19—C20	176.6 (2)
C5—C6—C7—C8	−178.7 (2)		

### Hydrogen-bond geometry (Å, °)

**Table:**

*D*—H···*A*	*D*—H	H···*A*	*D*···*A*	*D*—H···*A*
O2—H2···O1	0.84 (1)	1.73 (1)	2.561 (2)	173 (4)
N2—H1···N3	0.88 (1)	2.08 (1)	2.943 (3)	171 (3)

## References

[bb1] Agilent (2010). *CrysAlis PRO* Agilent Technologies, Yarnton, England.

[bb2] Barbour, L. J. (2001). *J. Supramol. Chem.* **1**, 189–191.

[bb3] Najafi, E., Amini, M. M. & Ng, S. W. (2010*a*). *Acta Cryst.* E**66**, m1276. 10.1107/S1600536810036706 PMC2983420 21587420

[bb4] Najafi, E., Amini, M. M. & Ng, S. W. (2010*b*). *Acta Cryst.* E**66**, m1277. 10.1107/S1600536810036718 PMC2983225 21587421

[bb5] Najafi, E., Amini, M. M. & Ng, S. W. (2011). *Acta Cryst.* E**67**, m1280. 10.1107/S1600536811032338 PMC3200945 22058876

[bb6] Sattarzadeh, E., Mohammadnezhad, G., Amini, M. M. & Ng, S. W. (2009). *Acta Cryst.* E**65**, m553. 10.1107/S1600536809014202 PMC2977600 21583786

[bb7] Sheldrick, G. M. (2008). *Acta Cryst.* A**64**, 112–122. 10.1107/S0108767307043930 18156677

[bb8] Westrip, S. P. (2010). *J. Appl. Cryst.* **43**, 920–925.

